# German Chamomile (*Matricaria chamomilla* L.) Flower Extract, Its Amino Acid Preparations and 3D-Printed Dosage Forms: Phytochemical, Pharmacological, Technological, and Molecular Docking Study

**DOI:** 10.3390/ijms25158292

**Published:** 2024-07-29

**Authors:** Oleh Koshovyi, Janne Sepp, Valdas Jakštas, Vaidotas Žvikas, Igor Kireyev, Yevhen Karpun, Vira Odyntsova, Jyrki Heinämäki, Ain Raal

**Affiliations:** 1 Institute of Pharmacy, Faculty of Medicine, University of Tartu, 50411 Tartu, Estonia; janne.sepp@ut.ee (J.S.); jyrki.heinamaki@ut.ee (J.H.); ain.raal@ut.ee (A.R.); 2 The Department of Clinical Pharmacology and Clinical Pharmacy, National University of Pharmacy, 61002 Kharkiv, Ukraine; ivkireev@ukr.net; 3 Institute of Pharmaceutical Technologies, Lithuanian University of Health Sciences, LT-44307 Kaunas, Lithuania; valdas.jakstas@lsmu.lt (V.J.); vaidotas.zvikas@lsmu.lt (V.Ž.); 4 Life Chemicals Inc., 02094 Kyiv, Ukraine; ekarpun@yahoo.com; 5 The Department of Pharmacognosy, Pharmacology, and Botany, Zaporizhzhia State Medical and Pharmaceutical University, 69035 Zaporizhzhia, Ukraine; odyntsova1505@gmail.com

**Keywords:** German chamomile (*Matricaria chamomilla* L.) plant extract, polyphenolic compounds, amino acids, lysine, β-alanine, analgesic activity, soporific activity, three-dimensional printing

## Abstract

German chamomile (*Matricaria chamomilla* L.) is an essential oil- containing medicinal plant used worldwide. The aim of this study was to gain knowledge of the phytochemical composition and the analgesic and soporific activity of *Matricaria chamomilla* L. (German chamomile) flower extract and its amino acid preparations, to predict the mechanisms of their effects by molecular docking and to develop aqueous printing gels and novel 3D-printed oral dosage forms for the flower extracts. In total, 22 polyphenolic compounds and 14 amino acids were identified and quantified in the *M. chamomilla* extracts. In vivo animal studies with rodents showed that the oral administration of such extracts revealed the potential for treating of sleep disorders and diseases accompanied by pain. Amino acids were found to potentiate these effects. Glycine enhanced the analgesic activity the most, while lysine and β-alanine improved the soporific activity. The molecular docking analysis revealed a high probability of γ-aminobutyric acid type A (GABAA) and N-methyl-D-aspartate (NMDA) receptor antagonism and 5-lipoxygenase (LOX-5) inhibition by the extracts. A polyethylene oxide (PEO)-based gel composition with the *M. chamomilla* extracts was proposed for preparing a novel 3D-printed dosage form for oral administration. These 3D-printed extract preparations can be used, for example, in dietary supplement applications.

## 1. Introduction

German chamomile (*Matricaria chamomilla* L., Asteraceae family) is an essential oil-containing medicinal plant widely used in folk and official medicine in Europe, America, and Asia [1,2,3]. *M. chamomilla* has been traditionally used for the treatment of gastrointestinal disorders [4], neuropsychiatric problems [5], skin and mouth diseases, common cold, and respiratory infections [6]. Chamomile remedies are widely used against pain and infections [3].

The essential oil components of *M. chamomilla* flowers have been intensely studied, and in previous research works from 22 to over 120 such constituents have been identified [2,3]. In addition to essential oil components, *M. chamomilla* extracts also contain polyphenolic compounds, such as phenolic and hydrocinnamic acids, coumarins, and flavonoids [3,7,8,9,10,11].

In both human patients and animal models, the therapeutic effect of chamomile remedies has been revealed on different diseases, such as gastrointestinal and skin, nervous, and cardiovascular ailments, metabolic disorders, eye dysfunctions, and allergies [3]. Among galenic chamomile medicines, the dry extract based on a chamomile tincture has been shown to possess the most significant soporific and analgesic activity [7]. Modifications of the extract’s biologically active substances can be used to enhance these effects. A well-known strategy is conjugating extract constituents with amino acids [12,13]. This strategy was used, for example, in the development of the antiherpetic drug Valtrex by synthesizing acyclovir with valine [14], and in the development of L-lysine aescinate by combining β-escin (chestnut triterpene saponins) with L-lysine [14,15].

Karpov and Yu [16] proved that arginine enhances the bioavailability (absorption rate) and stability of perindopril and also reduces side effects. Modifying acyclovir with valine formed a novel active substance—valacyclovir, which significantly increases systemic plasma levels of acyclovir. That improved patient convenience and clinical efficacy [17]. *Leonurus cardiaca* L. tincture was also combined with amino acids, which led to the discovery of novel extracts with enhanced anxiolytic activity [18]. Highbush blueberry (*Vaccinium corymbosum* L. Ericaceae) [13] and cranberry leaf extracts (*Vaccinium macrocarpon* Aiton, Ericaceae) [19] in combination with arginine have enabled the development of new active ingredients with promising hypoglycaemic and hypolipidemic activity. The abovementioned examples suggest the feasibility of this chamomile extract modification strategy in developing new active constituents.

Plant-origin medicines are characterized by a positive safety profile. In combinatorial drug therapy, plant-origin extract(s) and tincture(s) are combined with synthetic drug(s) to enhance the drug treatment and efficacy. To date, the galenic dosage forms of such plant-origin materials (i.e., tinctures, liquid extracts, teas, decoctions, etc.), however, have a low level of compliance and a lack of standardization. In this case, the pharmaceutical 3D printing of novel oral dosage forms could be a promising approach to enhance the efficacy and compliance of plant-origin remedies. The successful development of medicinal 3D-printed dosage forms for plant-origin materials (such as chamomile extracts) requires multidisciplinary expertise in pharmaceutical technology, polymer chemistry, engineering sciences, and pharmacognosy.

The aim of the study was three-fold: (1) to carry out research and gain knowledge of the phytochemical constituents, the analgesic and soporific activity of *M. chamomilla* flower extract and its amino acid preparations, (2) to predict the mechanisms of their effects by molecular docking, and (3) to develop aqueous gel formulations loaded with the extracts for semi-solid extrusion (SSE) 3D printing and to prepare novel 3D-printed oral dosage forms for the present chamomile extracts.

## 2. Results

The *M. chamomilla* dry extracts were brown powders with a specific smell. The loss on drying (LOD) data of the extracts were from 4.1% to 4.7%.

### 2.1. Phytochemical Study

**Figure 1:**
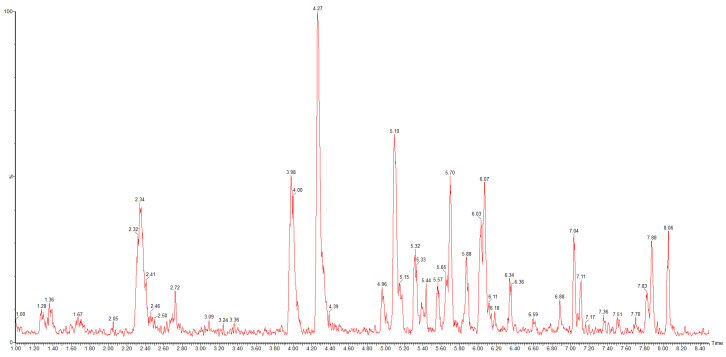
HPLC-MS scan chromatogram in negative ESI mode of ethanolic (Gch) German chamomile (*Matricaria chamomilla* L.) flower extract.

The main polyphenolic compounds of the dry *M. chamomilla* extract and its amino acid preparations were studied by a UPLC-MS/MS method, and the results are gained in Table 1 and Figure 1. The quantification of hydrocinnamic acids, flavonoids, and total polyphenolic compounds was also carried out by European Pharmacopoeia (Ph.Eur.) methods of spectrophotometry [20]. A total of 22 polyphenolic compounds two phenolic and seven hydroxycinnamic acids, and 13 flavonoids) were identified and quantified in the dry *M. chamomilla* extract and its amino acid preparations.

The amino acids in the dry *M. chamomilla* extract and its amino acid preparations were studied by a UPLC-MS/MS method and are presented in Table 2. A total of 14 amino acids were identified and quantified in the dry German chamomile extracts.

### 2.2. Pharmacological Research on Analgesic and Soporific Activity, and Molecular Docking Study

Chamomile galenic remedies are used in folk medicine for skin and dental diseases, as well as insomnia. Therefore, we were interested in investigating and proving the analgetic and soporific activity of the dry chamomile extract and its amino acid preparations. This study also enabled us to settle the influence of amino acids on the level of these effects.

#### 2.2.1. Analgesic Activity

The analgesic activity of the dry *M. chamomilla* extract and its amino acid preparations were studied with mice in a hot-plate test, and the results are shown in Table 3. The dry chamomile extracts impacted the thermal stimulus response. Some amino acids enhanced this extract’s effect and are compared to acetaminophen.

#### 2.2.2. Soporific Activity

The soporific activity of the *M. chamomilla* dry extract and its amino acid preparations was studied by fixing sleep duration and is presented in Table 4. The extracts prolonged the sleeping period. The results were compared with a group of rats which consumed sodium thiopental in a dose of 40 mg/kg and the animal group given the *M. chamomilla* dry extract to establish the influence of amino acids on this effect.

#### 2.2.3. Molecular Docking Study

Polyphenolic compounds are promising alternative agents with analgesic, anti-inflammatory, and sedative-hypnotic effects, particularly in targeting cyclooxygenases (COX-1/COX-2), mu-opioid/kappa-opioid/N-methyl-D-aspartate (NMDA)/γ-aminobutyric acid type A (GABAA) receptors, prostaglandin E synthase, and 5-lipoxygenase (5-LOX). Therefore, molecular docking of the identified polyphenolic compounds was carried out to predict their soporific and analgesic activity and the results are presented in Table S1.

### 2.3. Preparation of 3D-Printed Oral Dosage Forms with the M. chamomilla Dry Extract

The aqueous 12% polyethylene oxide (PEO) gels loaded with the German chamomile dry extract (0.5, 1.0 and 1.5 g in 10 g) were yellow-brownish viscous masses with a specific smell.

**Figure 2:**
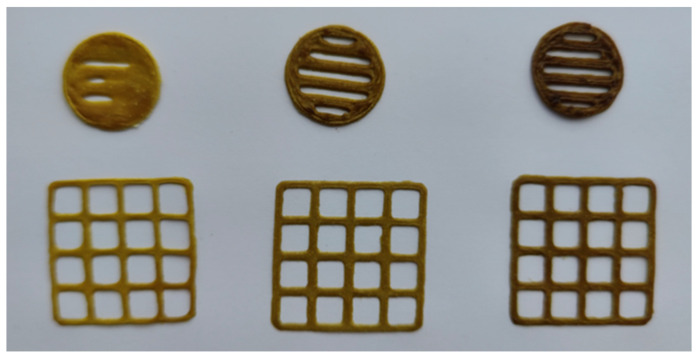
Photographs of the semi-solid extrusion (SSE) 3D-printed lattices and round-shaped discs. The constructs were printed based on the aqueous 12% polyethylene oxide (PEO) gel with the *M. chamomilla* dry extract.

The viscosity of the abovementioned gels with the German chamomile dry extract was investigated at a speed of 0.05 RPM and a shear rate of 0.100 1/s at room temperature 22 ± 2 °C (Table 5). The speed of the printing head was 0.5 mm/s, as was reported previously for PEO gels with plant extracts [19,21]. The other operating parameters of SSE 3D printing were also optimized [22] and implemented in our study for 3D printing of the gels with the *M. chamomilla* dry extract. Standard-size square-shaped 3D lattices (30 × 30 × 0.5 mm, six layers) and round-shaped discs (diameter of 20 mm, five layers) were printed for the verification of the feasibility of the aqueous PEO gels with the German chamomile dry extract for SSE 3D printing (Figure 2). The surface area of the 3D-printed lattices, average S_practical_/S_theoretical_ ratio of the lattices, and average mass of the scaffolds were calculated and the results are presented in Table 5.

## 3. Discussion

A total of 22 polyphenolic compounds were determined in the *M. chamomilla* extract and its amino acid preparations. These findings are in line with the results reported in previous studies [7]. We found that the native *M. chamomilla* extract (70% aqueous ethanol) contains hydroxycinnamic acids, such as chlorogenic acid, 3,5-dicaffeoylquinic acid, 4,5-dicaffeoylquinic acid, and 3,4-dicaffeoylquinic acid. This finding differed from the previously known results [8,23,24], where caffeic and ferulic acid were shown to be the main hydroxycinnamic acids in the *M. chamomilla* extract. Among flavonoids, luteolin and quercetin derivatives predominated; such constituents as luteolin-7-O-glucoside and isoquercitrin are the main ones. In the previous studies, the main flavonoids of *M. chamomilla* flowers were apigenin and luteolin, patuletin, and quercetin [23]. The significant amount of 3,4-dihydroxyphenylacetic acid in the *M. chamomilla* extracts could provide the analgesic activity of such plant extracts [25]. We found that the content of all the identified compounds in the amino acid preparations of *M. chamomilla* extracts is lower than in the native extract.

A total of 14 amino acids were found in the dry *M. chamomilla* extract, six of which are essential. Glutamic acid, proline, and threonine predominated.

Glutamic acid is the most abundant excitatory neurotransmitter in the vertebrate nervous system. It serves as the precursor for synthesising of the inhibitory gamma-aminobutyric acid (GABA) in GABAergic neurons [26,27]. Therefore, it could affect the soporific activity of the *M. chamomilla* dry extract. L-Proline is a weak agonist of the glycine receptor and of both NMDA and non-NMDA (AMPA/kainate) ionotropic glutamate receptors [28,29]. Threonine is an essential amino acid, which is used to synthesize glycine during the endogenous production of L-carnitine in the brain and liver [30,31]. It also regulates neurotransmission in the brain and helps fight depression. Lack of threonine contributes to the rapid development of fatigue [32].

Previous studies proved that chamomile extracts have anti-inflammatory and analgesic activity [1,7,33]. The modifications with amino acids provided some potentiation of the *M. chamomilla* extract analgesic activity. These were typical for the extracts modified with glycine. The extract preparations with phenylalanine, valine, and lysine at doses of 50 and 100 mg/kg showed a higher analgetic effect than the control group and the native *M. chamomilla* extract. However, the most promising one was the extract modified with glycine. At doses of 50 and 100 mg/kg, the activity of the extract with glycine even exceeded the analgesic effect of acetaminophen.

Chamomile tea has been used for ages in the treatment of sleep disorders [1,33]. In previous studies, we found that the *M. chamomilla* extracts had a sedative effect [7]. The most effective dose of the German chamomile extract was 50 mg/kg, which increased sleep duration by 117.3% [7]. We found that β-alanine and lysine exhibit a synergistic soporific effect with the *M. chamomilla* extract. Thus, at 25 mg/kg and 100 mg/kg doses, the lysine-modified extract increased sleep duration by 158.9 and 152.9% compared to the *M. chamomilla* extract at similar doses. The extract with β-alanine also prolonged the sleep period by 156.4% compared to the native extract at the same dose.

Previously, it was also reported that *M. discoidea* extracts in in vivo studies with rodents demonstrated analgesic activity and improvements in the sleep of animals [34], but these effects of *M. chamomilla* extract and its amino acid preparations are more intense and pronounced. Dry motherwort (*Leonurus cardiaca* L.) extracts also showed that glycine, valine, and arginine potentiate their anxiolytic activity [16]. However, for the chamomile ones, these amino acids did not similarly manifest themselves; in this case, modification with lysine and β-alanine turned out to be more promising.

It was reported [1,2,3] that polyphenolic compounds cause these effects. Essential oil [7,8] can also affect this, but its concentration in the extract is quite low. Using HPLC methods, we determined the main phenolic compounds and determined the amino acid composition of the extracts. Amino acids were added to the native extract in an amount that is much lower than their therapeutic dose. Thus, it is evident that the changes in the pharmacokinetics of the modified extracts resulted in the facilitated transport of such actives across tissue barriers followed by the higher activity of pain control mechanisms.

The main polyphenolics were preliminarily studied in silico, namely the binding interactions of ligands in the binding pockets of the selected proteins were further analysed using docking studies. But this approach does not overlook other major compounds and their combinations, which may have significant influence on total pharmacological effect.

We obtained binding affinity values in kcal/mol and CNN pose estimation for each of the eight proteins interacting with the 17 ligands via GNINA. As seen in Table S1, the highest value of affinity (kcal/mol) in relation to the comparison drugs was obtained for the rutin-bound mu-opioid receptor (−11.12 kcal/mol), luteolin 7,3′-diglucoside with GABAA receptor (−7.17 kcal/mol), and 3,5-dicaffeoylquinic acid with NMDA receptor (−10.70 kcal/mol). At the same time, cryptochlorogenic acid showed the highest stabilized interaction as assessed by the CNN pose with 5-lipoxygenase (0.8774).

It was determined that rutin is well integrated into the active site of the mu-opioid receptor (PBD ID 5C1M) due to the formation of hydrogen bonds with ASP147 (2.6 Å), HIS297 (2.2 Å), TYR148 (2.9 Å), LYS233 (2.8 Å) THR218 (3.0 Å), as well as the π-π stacking interaction between the π-electron cloud of the aromatic ring of 3,4-dihydroxyphenyl and 4-hydroxyphenyl TYR326 (Figure S1.1).

For 3,5-dicaffeoylquinic acid, the best binding pose was achieved with the NMDA receptor (Figure S1.2) through a series of hydrogen contacts of oxo-functional groups with GLN13 (2.2 Å), ALA206 (2.4 Å), SER180 (2.3 Å), SER179 (2.5 Å), VAL181 (2.2 Å), THR126 (1.9 Å), ASN128 (2.4, 2.0 Å). A similar binding mode is presented in the article [35].

By contrast, AKBA, which, due to its skeleton, forms three hydrogen bonds with AGR156, VAL128, and HIS148 [36], has a cryptochlorogenic acid (Figure S1.3), and a different H-binding profile with the best CNN pose score of 0.8774 points. The difference was hydrogen contacts between the VAL110, GLU134, ARG68, AGR101 residues.

The determined affinity index for luteolin 7,3′-diglucoside for the GABAA receptor was −7.17 kcal/mol. The ligand exhibited a position characterized by two classical hydrogen bonds with ASP282 (1.9, 2.7 Å) through hydroxyl groups (Figure S1.4). ARG269 also formed hydrocarbon bonds through the available amino groups.

Based on the results of docking and analysis of binding modes, it can be concluded that the present phytochemicals can most likely exert their anti-inflammatory, sedative, and analgesic activity by inhibiting the studied enzymes and receptors. However, further molecular dynamics studies are necessary to finally confirm the stability of the formed complexes and mechanisms of their activity. They also should be studied both in vitro and in vivo to assess their safety and efficacy as therapeutic agents.

Previously, the results of the molecular docking analyses of the identified BASs of *M. discoidea* demonstrated a high probability of COX-1,2 inhibition and NMDA receptor antagonism [34]. This corresponds to the results obtained for *M. chamomilla* extract.

Therefore, the phytochemicals found in *M. chamomilla* flowers proved to be promising candidates for the development of analgesic drugs. However, further research work and development are needed to adopt the use of such new potent anti-inflammatory agents and hypnotics for therapeutic applications.

The aqueous PEO gels mixed with the *M. chamomilla* extract showed a homogeneous structure. The aqueous 12% PEO gel was a feasible platform for printing gels with the *M. chamomilla* extract at concentrations ranging from 0.5 to 1.5 g per 10 mL. The corresponding SSE 3D-printed scaffolds were uniform in shape and size (Figure 1). By visual inspection, dissolving the printed samples in purified water at a room temperature (22 ± 2 °C) without mixing, it was settled that 3D-printed dosage forms were completely disintegrated within 20–25 min, thus suggesting that they could be used as an immediate-release oral delivery system for the present plant extract.

## 4. Materials and Methods

### 4.1. Chemicals

Deionized water was obtained by using a Millipore Simplicity UV station (Merck Millipore, Burlington, MA, USA). Ethanol, formic acid, and acetonitrile originated from VWR (Radnor, PA, USA). DL-valine, glycine, phenylalanine (Acros Organics, Geel, Belgium), β-alanine (OstroVit, Zambrow, Poland), L-arginine, L-lysine (Fits OÜ, Tallinn, Estonia), Tween-80 (Ferak, Berlin, Germany), and aluminium chloride (Sigma-Aldrich, St. Louis, MO, USA), rutin, chlorogenic and gallic acids (Carl Roth, Karlsruhe, Germany) were used in our experiments.

### 4.2. Plant Material

*Matricaria chamomilla* L. flowers were produced by MK Loodusravi OÜ (Venevere village, Põhja-Sakala municipality, Viljandi County, Estonia (58.598228 N, 25.704950 E)) and packed in an airtight plastic bag. The identity of the raw material was confirmed by Prof. Ain Raal, Institute of Pharmacy, University of Tartu, Tartu, Estonia [20,37]. The *M. chamomilla* flowers raw material met the European Pharmacopeia’s requirements [20]. Its loss on drying was 6.8% [20].

### 4.3. Preparation of Extracts

The dried *M. chamomilla* flowers (500.0 g) were extracted with 70% aqueous ethanol solution (3000 mL) by macerating at ambient room temperature for one day. The extraction was repeated twice with the same solvent (1000.0 mL at each stage). The liquid extracts were combined, kept for sedimentation for two days, and filtrated. The six amino acids of interest, arginine (2.84 g), phenylalanine (2.68 g), β-alanine (1.44 g), glycine (1.23 g), valine (1.90 g), and lysine (2.37 g), were added in a three-fold equimolar amount to the total polyphenolic compounds in terms of gallic acid to the six portions of the liquid extract (300 mL each one). The resulting solutions were kept overnight at 22 ± 2 °C.

The native liquid extract and its solutions with amino acids were separately evaporated in a rotary vacuum evaporator (vacuum 150 mbar, rotation 80 rpm, heating bath 80 °C) to form thick extracts, which were then lyophilized in a SCANVAC COOLSAFE 55-4 Pro (LaboGene ApS, Lillerød, Denmark) apparatus. Finally, the seven dry extracts were obtained: the dried *M. chamomilla* flower extract (Gch) and its amino acid preparations with arginine (Gch-Arg), phenylalanine (Gch-Phe), β-alanine (Gch-β-Ala), glycine (Gch-Gly), valine (Gch-Val), and lysine (Gch-Lys).

### 4.4. Spectrophotometric Assay of Main Groups of Phytochemicals

The assay of flavonoids, hydroxycinnamic acids, and total phenols in the *M. chamomilla* extract and its amino acid preparations were carried out with a Shimadzu UV-1800 spectrophotometer (Shimadzu Corporation, Tokyo, Japan). Flavonoids were calculated in terms of rutin. Optical density was measured at 417 nm after the reaction with aluminium chloride [20,38]. Hydroxycinnamic acids were estimated after adding sodium molybdate and sodium nitrite and measuring optical density at 525 nm. The calculation was performed in terms of chlorogenic acid [20]. Total polyphenolic compounds were assayed with gallic acid at 270 nm [39]. The experiments were repeated three times.

### 4.5. Assay of Polyphenolic Compounds by UPLC-MS/MS

*M. chamomilla* flower samples were analysed with a UPLC-MS/MS system to determine the quantitative composition of polyphenolic compounds. The compounds of interest were separated from the plant matrix using an Acquity H-class UPLC system (Waters, Milford, MA, USA) coupled with a YMC Triart C18 (100 × 2.0 mm 1.9 µm) column. Column temperature was maintained at 40 °C. The mobile phase was supplied at 0.5 mL/min flow rate consisting of an aqueous solution of formic acid (0.1%) as eluent A, and MS-grade acetonitrile as eluent B. The following linear gradient was applied: from 0 to 1 min, constant flow at 95% of solvent A; 1 to 5 min, linear increase in solvent B to 30%; 5 to 7 min, to 50%; 7.5 to 8 min, wash column with 100% solvent B; return to initial conditions for a total analysis time of 10 min. Mass spectrometric data were acquired with a triple-quadrupole tandem mass spectrometer (Xevo, Waters, USA) working in negative electrospray ionization (ESI) mode. The following parameters for the MS/MS acquisition were set: the capillary voltage was set to −2 kV, cone voltage set at 30 V, desolvation gas (nitrogen) was heated to 400 °C and supplied at a flow rate of 700 L/h, the curtain gas (nitrogen) flow was supplied at 20 L/h. The temperature of the ion source was maintained at 150 °C. Analytical-grade standards were used to identify polyphenolic compounds in *M. chamomilla* flowers by comparing their MS/MS spectral data and retention times. Linear regression fit and standard dilution methods were used for the quantitative determination of polyphenolic compounds [40,41].

### 4.6. Assay of Amino Acids by UPLC-MS/MS

Analysis of amino acids in *M. chamomilla* flowers was carried out on Acquity H-class UPLC system (Waters, Milford, MA, USA) equipped with a Xevo TQD mass spectrometer (Waters, Milford, MA, USA). One microliter of extracts was injected into a BEH Amide (150 mm × 2.1 mm, 1.7 µm) column (Waters, Milford, MA, USA) with column temperature set at 25 °C. The mobile phase consisting of an aqueous solution of 10 mmol ammonium formate with 0.125% formic acid (eluent A) and acetonitrile (eluent B) was delivered at 0.6 mL/min. The gradient elution method with the following settings was applied: 0 min to 1 min, 95% B; 1–3.9 min, 70% B; 3.9–5.1 min, 30% B; 5.1–6.4 min, the column was flushed with 70% of eluent A; at 6.5 min, the gradient was returned to the initial composition for a total run time of 10 min. Mass spectrometer conditions were set as follows: positive electrospray ionization was set to +3.5 kV, cone voltage set at 30 V, desolvation gas flow at 800 L/h, and temperature at 400 °C. The ion source temperature was set at 120 °C. Peak assignment and identification of amino acids in *M. chamomilla* flower extracts were carried out while comparing their retention times and MS/MS data with those of analytical-grade standards. Linear regression fit models were obtained using the standard dilution method to quantify amino acids.

### 4.7. Pharmacological Study

The analgesic and soporific activity was studied with rodents (rats and mice) in compliance with the rules of the European Convention for the Protection of Vertebrate Animals Used for Experimental and Other Scientific Purposes (Strasbourg, 1986), Directive 2010/63/EU of the European Parliament and of the Council of the European Union (2010) on the protection of animals used for scientific purposes, the Order of the Ministry of Health of Ukraine No. 944 “On Approval of the Procedure for Preclinical Study of Medicinal Products and Examination of Materials for Preclinical Study of Medicinal Products” (2009), and the Law of Ukraine No. 3447-IV “On the protection of animals from cruel treatment” (2006) [42,43,44,45,46]. The research was approved by the Bioethics Commission of the National University of Pharmacy, Ukraine (protocol №4 from 3 October 2023).

#### 4.7.1. Analgesic Activity

The analgesic activity of the *M. chamomilla* extract and its amino acid preparations was studied with mice (20–40 g) in comparison with acetaminophen (paracetamol-Zdorovye, 500 mg capsules, pharmaceutical company Zdorovye, Kharkiv, Ukraine).

Before the test, the animals did not have access to food for 2 h. The experimental groups (six mice in each group) were formed randomly: Group 1—intact animals (1 mL 0.9% solution of NaCl per 100 g of body weight); Groups 2–4—animals treated with extract Gch at doses of 25, 50, and 100 mg/kg; Groups 5–7—animals treated with extract Gch-Arg at doses of 25, 50, and 100 mg/kg; Groups 8–10—animals treated with extract Gch-Phe at doses of 25, 50, and 100 mg/kg; Groups 11–13—animals treated with extract Gch-β-Ala at doses of 25, 50, and 100 mg/kg; Groups 14–16—animals treated with extract Gch-Gly at doses of 25, 50, and 100 mg/kg; Groups 17–19—animals treated with extract Gch-Val at doses of 25, 50, and 100 mg/kg; Groups 20–22—animals treated with extract Gch-Lys at doses of 25, 50, and 100 mg/kg; and control group (CG) 23—animals treated with acetaminophen at a dose of 50 mg/kg.

The agents were administered intragastrically, and subsequently, the animal was carefully placed on a hot plate (55 °C) for 30 min. The duration of the mouse’s stay on the hot plate (in seconds) before the beginning of protective reflexes was chosen as an indicator of pain sensitivity. The time during which the animals were on the hot plate did not exceed 60 s. After the agent consumption, the mice were examined at regular intervals at 0.5, 1, 2, 3, and 4 h. The analgesic effect was revealed when the latent period of response after the administration of the agent significantly increased in the control group [7,47,48].

#### 4.7.2. Soporific Activity

The soporific activity of the *M. chamomilla* extract and its amino acid preparations was studied with white rats (190–280 g). The reference drugs were sodium thiopental lyophilizate (for injection solution, PLC Kiivmedpreparat, Kyiv, Ukraine), and AN NATUREL valerian syrup (LLC Beauty and Health, Kharkiv, Ukraine).

The experimental groups (six mice in each group) were formed randomly: Group 1—intact animals (1 mL 0.9% solution of NaCl per 100 g of body weight); Groups 2–4—animals treated with extract Gch at doses of 25, 50, and 100 mg/kg; Groups 5–7—animals treated with extract Gch-Arg at doses of 25, 50, and 100 mg/kg; Groups 8–10—animals treated with extract Gch-Phe at doses of 25, 50, and 100 mg/kg; Groups 11–13—animals treated with extract Gch-β-Ala at doses of 25, 50, and 100 mg/kg; Groups 14–16—animals treated with extract Gch-Gly at doses of 25, 50, and 100 mg/kg; Groups 17–19—animals treated with extract Gch-Val at doses of 25, 50, and 100 mg/kg; Groups 20–22—animals treated with extract Gch-Lys at doses of 25, 50, and 100 mg/kg; and control group (CG) 23—animals treated with sodium thiopental at a dose of 40 mg/kg; control group (CG valerian) 24—animals treated with valerian syrup at a dose of 2.14 mg/kg. The duration of sleep was determined by the time period for which the rats were in a lateral position [7,34,49,50].

#### 4.7.3. Molecular Docking Analysis

Selection of ligands. The following constituents derived from *M. chamomilla* were used: 4,5-dicaffeoylquinic acid (PubChem CID: 5281780), 3,4-dicaffeoylquinic acid (PubChem CID: 6474309), luteolin 7,3′-diglucoside (PubChem CID: 44258089), 3,5-dicaffeoylquinic acid (PubChem CID: 13604688), rutin (PubChem CID: 5280805), luteolin 7-O-glucoside (PubChem CID: 5280637), isorhamnetin (PubChem CID: 5281654), luteolin (PubChem CID: 5280445), cryptochlorogenic acid (PubChem CID: 9798666), chlorogenic acid (PubChem CID: 1794427), hyperoside (PubChem CID: 5281643), isorhamnetin-3-glucoside (PubChem CID: 6455477), luteolin-4-O-glucoside (PubChem CID: 12304738), neochlorogenic acid (PubChem CID: 5280633), caffeic acid (PubChem CID: 689043), 3,4-dihydroxyphenylacetic acid (PubChem CID: 547), and vanillic acid (PubChem CID: 8468). The abovementioned constituents were converted to 2DSDF format and subjected to minimization by using the Open Babel tool for facilitating the determination of their binding affinity to specific targets.

Preparation of targeted proteins. The three-dimensional arrangements of various protein complexes were retrieved from the RCSB Protein Data Bank in PDB format, including the mu-opioid receptor-Gi protein complex (PDB: 5C1M) [51], SC-558 bound at the COX-2 active site (PDB: 1CX2) [52], COX-1 complexed with ibuprofen (PDB: 1EQG) [53], crystal structure of the NMDA receptor GluN1 (PDB: 4KFQ) [34], nanobody-stabilized active state of the kappa-opioid receptor (PDB: 6B73) [54], human hematopoietic prostaglandin D synthase (PDB: 2CVD) [55], stable-5-lipoxygenase bound to AKBA (PDB: 6NCF) [55], and human GABAA receptor (PDB: 6X3X) [56].

Docking Analysis. Docking studies were performed for the most active compounds which had the potential to be the best inhibitors of selected proteins in the binding site of enzymes using GNINA [57], which utilizes an ensemble of convolutional neural networks (CNNs) as a scoring function. The scores include binding affinities (kcal/mol), and CNN pose scores (the probability that the pose has a low root mean square deviation from the binding pose) predicted by GNINA. Ligand molecules had ten degrees of freedom provided. Using an autobox ligand, a grid box with an active site in the centre was built. BIOVIA Discovery Studio Visualizer 2020 [58] and Pymol 2.6 (Schrödinger LLC., New York, NY, USA) were eventually accelerated to evaluate the docking sites for the potential linking approaches.

### 4.8. Three-Dimensional Printing of M. chamomilla Extract

Polyethelen oxide (PEO) (MW approx. 900,000, Sigma-Aldrich, USA) at concentrations of 12% were implemented for preparing aqueous gels with *M. chamomilla* extract for the SSE 3D printing. For this, 1.2 g of PEO was mixed in 10 mL of purified water at least for 13–15 h at an ambient room temperature [19,21,22]. Tween 80 (Laborat GMBH, Berlin, Germany) was used to create a more stable and homogenised system and enhance the release of *M. chamomilla* extracts from the 3D-printed scaffolds [19,21]. The *M. chamomilla* extract (0.5, 1.0, and 1.5 g) and Tween 80 as a surface-active agent (0.5 g) were added to 12% PEO gel. The gel viscosity was measured at room temperature (22 ± 2 °C) with a Physica MCR 101 rheometer (Anton Paar, Graz, Austria).

The 12% PEO gels loaded with the *M. chamomilla* extract were directly printed using a bench-top semi-solid extrusion (SSE) 3D printing system (System 30 M, Hyrel 3D, Norcross, GA, USA). The printing control software of the SSE 3D printer was Repetrel, Rev3.083_K, Hyrel 3D, USA. The following parameters were used: printing head speed—0.5 mm/s; blunt needle (gauge, 21G); no heating of syringe content or printed plate.

A model 4 × 4 grid lattice (30 × 30 × 0.5 mm) and the round shapes (20 mm in diameter) were designed with Autodesk 3ds Max Design 2017 software (Autodesk Inc., San Francisco, CA, USA) and FreeCAD software (version 0.19/release date 2021) [59]. Six layers were printed to prepare the lattices, and five layers for the round-shaped scaffolds.

Lattice weight and area measurements were determined for the evaluation of 3D printability. The 3D-printed lattice theoretical surface area was 324 mm^2^. It was compared with the corresponding areas of experimental 3D-printed lattices, and their ratios were calculated [21,22]. The photographs were analysed with ImageJ (National Institute of Health, Bethesda, MD, USA) image analysis software (version 1.51k). The weights of the 3D-printed preparations were determined with an analytical scale (Scaltec SBC 33, Scaltec, Germany).

### 4.9. Statistical Analysis

The average value was calculated for at least three measurements in the phytochemical study and six in the pharmacological study. The values of confidence intervals were calculated using the Student’s criterion. The data are presented as the mean ± SD [20,60].

## 5. Conclusions

The present study showed with animal models (rats and mice) the potential of the *M. chamomilla* extract and its amino acid preparations in treating sleep disorders and diseases accompanied by pain. A total of 22 polyphenolic compounds and 14 amino acids were identified and quantified in the extracts. It is possible that the targeted analysis chosen and conducted in the present study masked the presence of other bioactive compounds, which may indeed be present in these chamomile extracts and for which an untargeted analysis could also provide further information for their quantification and evaluation of their contribution in the observed biological activities. Nevertheless, the answer to this scientific question may provide future perspectives for further studying this natural source for other bioactive compounds too. The performance and efficacy of the extract can be additionally augmented by conjugation with amino acids. Glycine potentiates analgesic activity, while lysine and β-alanine preferably improve the soporific activity of the *M. chamomilla* extract. The molecular docking analysis revealed a high probability of COX-1,2 inhibition and GABAA and NMDA receptor antagonism by the *M. chamomilla* extract constituents. The formulation of novel 3D-printed oral dosage forms for the extracts is a promising approach for preparing the medicinal or dietary supplement products of such extracts.

## Figures and Tables

**Table 1:** Content of polyphenolic compounds in the *M. chamomilla* extract.

Substance		Retention Time, min	Content in the Extract						
			Gch [7]	Gch-Arg	Gch-Phe	Gch-β-Ala	Gch-Gly	Gch-Val	Gch-Lys
	UPLC-MS/MS, µg/g of dry extract								
Neochlorogenic acid		2.72	444.94 ± 20.16	329.21 ± 4.47	322.26 ± 8.51	373.81 ± 7.33	385.18 ± 13.11	382.70 ± 27.35	359.65 ± 12.79
Luteolin		7.15	310.93 ± 22.73	270.78 ± 15.81	320.87 ± 9.72	283.67 ± 8.20	309.48 ± 12.86	320.93 ± 27.22	301.31 ± 6.67
Isoquercitrin		5.49	921.16 ± 85.20	774.55 ± 41.11	850.92 ± 85.24	872.54 ± 34.08	880.89 ± 79.41	894.15 ± 43.80	838.97 ± 25.75
Cryptochlorogenic acid		3.86	80.74 ± 13.48	62.26 ± 9.76	70.97 ± 10.16	58.20 ± 5.03	62.47 ± 5.47	75.10 ± 20.95	55.98 ± 12.71
Luteolin-4-O-glucoside		6.03	45.11 ± 3.67	37.98 ± 1.88	49.59 ± 4.86	44.06 ± 3.86	41.68 ± 3.82	49.25 ± 6.75	43.68 ± 6.82
Chlorogenic acid		3.98	11,742.31 ± 376.34	8984.21 ± 397.30	9532.56 ± 179.37	10,341.94 ± 211.50	10,567.30 ± 220.17	10,014.55 ± 167.55	9342.78 ± 355.66
Quercetin		7.17	172.15 ± 12.01	138.19 ± 1.54	162.89 ± 8.50	149.96 ± 6.55	156.29 ± 6.19	149.27 ± 17.77	141.29 ± 6.20
Isorhamnetin-3-O-rutinoside		5.86	15.40 ± 1.60	12.03 ± 1.51	11.58 ± 1.28	12.78 ± 1.05	15.18 ± 1.55	14.68 ± 0.98	12.94 ± 1.50
Isorhamnetin-3-glucoside		6.12	410.75 ± 52.07	355.44 ± 24.71	402.53 ± 13.93	385.13 ± 32.20	448.75 ± 38.82	429.60 ± 16.85	406.41 ± 15.58
Luteolin-3,7-diglucoside		5.02	20.72 ± 1.88	15.60 ± 0.98	24.16 ± 0.70	19.03 ± 4.42	21.86 ± 1.36	20.04 ± 2.27	22.53 ± 6.19
Vanillic acid		4.22	86.58 ± 5.54	64.91 ± 5.23	69.80 ± 7.89	90.63 ± 2.98	88.66 ± 3.46	79.04 ± 3.04	85.28 ± 12.40
Caffeic acid		4.39	43.04 ± 3.22	40.58 ± 3.01	45.80 ± 6.49	47.91 ± 7.11	37.44 ± 4.50	36.52 ± 6.60	45.18 ± 4.35
3,4-Dihydroxyphenylacetic acid		2.34	184.05 ± 13.38	149.40 ± 12.77	145.05 ± 5.55	180.78 ± 11.94	154.06 ± 3.07	170.92 ± 4.40	151.33 ± 8.57
Isorhamnetin		7.95	125.32 ± 12.71	92.98 ± 5.25	116.50 ± 7.41	106.09 ± 5.58	101.81 ± 1.42	112.95 ± 3.76	102.56 ± 5.78
Apigenin		7.70	578.65 ± 63.91	462.21 ± 23.88	537.43 ± 47.49	506.71 ± 11.63	502.22 ± 3.14	549.22 ± 26.09	493.34 ± 29.54
Kaempherol-3-O-glucoside		5.94	50.76 ± 2.10	40.41 ± 3.67	49.94 ± 3.18	50.78 ± 5.32	55.22 ± 3.52	58.84 ± 2.27	48.15 ± 2.58
Rutin		5.39	126.49 ± 5.73	104.66 ± 3.94	102.88 ± 7.79	113.02 ± 14.86	109.13 ± 10.54	107.17 ± 10.24	99.26 ± 7.71
Hyperoside		5.44	366.82 ± 21.21	308.22 ± 21.78	315.79 ± 16.66	357.39 ± 12.20	387.16 ± 16.43	345.88 ± 21.19	347.21 ± 19.20
Luteolin-7-O-glucoside		5.56	1061.82 ± 83.68	874.36 ± 62.18	975.10 ± 57.44	1031.03 ± 73.93	1094.50 ± 72.63	1021.56 ± 59.44	1070.74 ± 37.66
4.5-Dicaffeoylquinic acid		5.70	4912.17 ± 416.85	3844.32 ± 149.55	4375.54 ± 208.35	4320.08 ± 175.34	4671.16 ± 242.58	4336.20 ± 352.82	4227.72 ± 365.27
3.5-Dicaffeoylquinic acid		6.07	2512.69 ± 213.23	1966.47 ± 76.50	2238.19 ± 106.57	2209.83 ± 89.69	2389.41 ± 124.08	2218.07 ± 180.47	2162.58 ± 186.85
3.4-Dicaffeoylquinic acid		5.88	5152.17 ± 437.22	4032.15 ± 156.86	4589.32 ± 218.53	4531.15 ± 183.91	4899.39 ± 254.43	4548.06 ± 370.05	4434.28 ± 383.12
	Spectrophotometry, %								
Total polyphenolic compounds			9.70 ± 0.52	7.87 ± 0.41	8.50 ± 0.34	9.40 ± 0.62	7.43 ± 0.59	8.29 ± 0.53	7.49 ± 0.09
Hydrocinnamic acids			3.47 ± 0.15	2.75 ± 0.29	3.28 ± 0.33	3.20 ± 0.14	2.64 ± 0.21	2.94 ± 0.31	2.50 ± 0.32
Flavonoids			9.92 ± 0.32	7.16 ± 0.38	8.50 ± 0.37	7.95 ± 0.09	7.55 ± 0.32	7.71 ± 0.77	7.26 ± 0.57

Notes: Gch—the dry *M. chamomilla* extract, obtained with 70% aqueous ethanol solution, and its amino acid preparations with arginine (Gch-Arg), phenylalanine (Gch-Phe), β-alanine (Gch-β-Ala), glycine (Gch-Gly), valine (Gch-Val), and lysine (Gch-Lys).

**Table 2:** Content of amino acids in the *M. chamomilla* extracts by UPLC-MS/MS.

Substance	Content in the Extract, mg/g						
	Gch	Gch-Arg	Gch-Phe	Gch-β-Ala	Gch-Gly	Gch-Val	Gch-Lys
Alanine (Ala)	2.90 ± 0.10	2.27 ±0.10	2.10 ± 0.07	2.59 ± 0.14	2.61 ± 0.23	2.59 ± 0.15	2.31 ± 0.11
Arginine (Arg)	1.34 ± 0.27	105.99 ± 2.03	3.09 ± 0.39	1.33 ± 0.16	1.03 ± 0.49	0.83 ± 0.07	7.48 ± 0.58
Aspartic acid (Asn)	1.44 ± 0.13	1.14 ± 0.04	1.10 ± 0.9	1.31 ± 0.14	0.99 ± 0.05	1.08 ± 0.05	1.20 ± 0.28
Glutamic acid (Glu)	2.13 ± 0.14	1.61 ± 0.06	1.73 ± 0.16	1.94 ± 0.30	2.04 ± 0.15	1.87 ± 0.16	2.02 ± 0.36
Glycine (Gly)	0.38 ± 0.01	0.37 ± 0.01	0.36 ± 0.01	0.32 ± 0.02	107.30 ± 8.80	0.92 ± 0.09	0.46 ± 0.07
Isoleucine (Ile)	1.76 ± 0.08	1.35 ± 0.04	1.43 ± 0.06	1.51 ± 0.07	1.43 ± 0.15	1.51 ± 0.09	1.32 ± 0.22
Leucine (Leu)	1.77 ± 0.11	1.37 ± 0.19	1.35 ± 0.14	1.53 ± 0.37	1.35 ± 0.43	3.07 ± 0.31	1.22 ± 0.09
Lysine (Lys)	1.10 ± 0.02	0.58 ± 0.09	0.69 ± 0.04	0.90 ± 0.01	0.90 ± 0.04	0.81 ± 0.02	298.56 ± 11.13
Phenylalanine (Phe)	1.07 ± 0.07	0.84 ± 0.08	181.68 ± 17.38	1.98 ± 0.69	0.96 ± 0.06	1.05 ± 0.15	0.74 ± 0.08
Proline (Pro)	2.70 ± 0.20	2.21 ± 0.02	2.25 ± 0.18	2.48 ± 0.25	2.56 ± 0.09	3.52 ± 0.19	2.63 ± 0.17
Serine (Ser)	1.96 ± 0.27	1.60 ± 0.04	1.45 ± 0.19	1.66 ± 0.06	1.77 ± 0.09	1.51 ± 0.09	1.61 ± 0.09
Threonine (Thr)	2.19 ± 0.22	1.81 ± 0.30	1.75 ± 0.13	1.94 ± 0.132	2.84 ± 0.09	2.06 ± 0.48	1.96 ± 0.19
Tyrosine (Tyr)	1.13 ± 0.13	0.85 ± 0.06	0.98 ± 0.01	1.02 ± 0.09	1.05 ± 0.05	1.00 ± 0.09	1.06 ± 0.11
Valine (Val)	1.52 ± 0.20	1.29 ± 0.11	1.34 ± 0.20	1.53 ± 0.15	1.47 ± 0.14	140.05 ± 7.33	1.86 ± 0.17
β-Alanine (β-Ala)	0	0	0	138.14 ± 9.77	0	0	0
Sum of amino acids	23.40	123.26	201.29	160.18	128.30	161.86	324.43

**Table 3:** Analgesic activity of the *M. chamomilla* extract and its amino acid preparations.

Phytosubstance	Group	Dose (mg/kg)	The Time of Response (s)/Analgesic Effect (%) in Comparison to (Reference Drug) and [Control]					
			Before	After Administration in				
				30 min	60 min	120 min	180 min	240 min
Control	1		6.84 ± 0.47	7.20 ± 0.29	7.10 ± 0.61	7.08 ± 0.27	7.15 ± 0.65	6.73 ± 0.94
Gch	2	25	7.93 ± 0.29	9.63 ± 0.54 [34%] (−8%) *	9.97 ± 0.60 [40%] (−4%) *	8.65 ± 0.48 [22%] # (−18%) *	7.98 ± 0.12 [12%] (−16%)	8.43 ± 0.21 [25%] (1%)
	3	50	7.68 ± 0.20	11.43 ± 0.85 [59%] # (9%)	11.83 ± 0.77 [67%] # (14%)	11.72 ± 0.73 [65%] # (11%)	11.13 ± 0.73 [56%] # (18%)	8.67 ± 0.31 [29%] # (4%)
	4	100	8.46 ± 0.42	12.50 ± 0.36 [74%] # (20%)	12.52 ± 0.31 [76%] # (21%) *	12.47 ± 0.30 [76%] # (18%) *	9.63 ± 0.50 [35%] # (2%)	9.02 ± 0.39 [34%] # (8%)
Gch-Arg	5	25	7.39 ± 0.31	8.36 ± 0.45 [16%] # (−20%) *	8.12 ± 0.60 [14%] (−22%) *	7.86 ± 0.41 [11%] (−25%) *	8.93 ± 0.33 [25%] # (−6%)	8.24 ± 0.52 [21%] (−1%)
	6	50	7.52 ± 0.16	8.40 ± 0.11 [17%] (−20%)	8.52 ± 1.11 [20%] (−18%)	8.85 ± 0.93 [25%] (−16%)	9.00 ± 0.99 [26%] (−5%)	7.82 ± 0.51 [16%] (−6%)
	7	100	7.63 ± 0.41	8.43 ± 0.63 [17%] (−19%)	8.48 ± 0.77 [19%] (−18%)	9.20 ± 0.53 [30%] # (−13%)	9.05 ± 0.80 [27%] (−4%)	7.45 ± 0.47 [11%] (−11%)
Gch-Phe	8	25	7.54 ± 0.31	8.04 ± 0.42 [12%] (−23%) *	8.51 ± 0.49 [20%] (−18%) *	9.04 ± 0.23 [28%] # (−14%)	9.10 ± 0.16 [27%] # (−4%)	8.81 ± 0.60 [31%] (6%)
	9	50	7.35 ± 0.26	9.58 ± 0.86 [33%] # (−8%)	10.67 ± 0.95 [50%] # (−3%)	10.37 ± 0.86 [46%] # (−2%)	9.70 ± 0.68 [36%] # (−3%)	8.12 ± 0.51 [21%] (−3%)
	10	100	7.43 ± 0.57	8.65 ± 0.68 [20%] # (−17%)	9.13 ± 0.65 [29%] # (−12%)	10.00 ± 0.50 [41%] # (−5%)	9.88 ± 0.51 [38%] # (5%)	8.03 ± 0.68 [19%] (−4%)
Gch-β-Ala	11	25	7.47 ± 0.27	8.79 ± 0.53 [22%] # (−16%) *	9.79 ± 0.72 [38%] # (−6%)	8.71 ± 0.44 [23%] # (−17%) *	8.73 ± 0.68 [22%] (−8%)	7.83 ± 0.41 [16%] (−6%)
	12	50	7.60 ± 0.16	8.33 ± 1.00 [16%] (−20%)	8.92 ± 1.01 [26%] (−14%)	8.93 ± 0.87 [26%] # (−15%)	9.17 ± 0.75 [28%] # (−3%)	7.98 ± 0.25 [19%] (−4%)
	13	100	7.59 ± 0.39	8.55 ± 0.58 [19%] (−18%)	8.53 ± 0.68 [20%] (−18%)	8.55 ± 0.60 [21%] (−19%)	8.47 ± 0.37 [18%] (−10%)	8.33 ± 0.29 [24%] (0%)
Gch-Gly	14	25	7.47 ± 0.27	9.86 ± 0.60 [37%] # (−6%)	10.46 ± 0.84 [47%] # (1%)	9.86 ± 0.49 [39%] # (−6%)	10.11 ± 0.58 [41%] # (7%)	9.89 ± 0.72 [47%] # (18%) *
	15	50	7.60 ± 0.16	10.02 ± 0.67 [39%] # (−4%)	10.35 ± 0.67 [46%] # (−0.3%)	10.38 ± 0.59 [47%] # (−1%)	10.00 ± 0.59 [40%] (6%)	9.46 ± 0.16 [40%] (13%)
	16	100	7.59 ± 0.39	11.25 ± 0.59 [56%] # (8%)	11.40 ± 0.66 [61%] # (10%)	11.68 ± 0.65 [65%] # (11%)	11.23 ± 0.64 [57%] # (19%) *	10.75 ± 0.45 [60%] # (29%) *
Gch-Val	17	25	7.50 ± 0.28	8.20 ± 0.49 [14%] (−22%) *	8.45 ± 0.48 [19%] (−19%) *	8.70 ± 0.53 [23%] # (−17%)	8.35 ± 0.33 [17%] (−12%)	7.52 ± 0.41 [12%] (−10%)
	18	50	7.53 ± 0.16	9.23 ± 0.83 [28%] # (−12%)	9.67 ± 0.83 [36%] # (−7%)	10.00 ± 0.81 [43%] # (−4%)	10.23 ± 0.73 [43%] # (8%)	9.60 ± 0.60 [43%] # (15%)
	19	100	7.56 ± 0.38	8.87 ± 0.57 [23%] # (−15%)	9.50 ± 0.55 [34%] # (−9%)	9.93 ± 0.54 [40%] # (−6%)	10.15 ± 0.40 [42%] # (7%)	9.33 ± 0.51 [39%] # (12%)
Gch-Lys	20	25	7.48 ± 0.29	9.83 ± 0.50 [37%] # (−6%)	10.12 ± 0.47 [42%] # (−3%)	9.73 ± 0.51 [37%] # (−8%)	9.52 ± 0.42 [33%] # (1%)	9.13 ± 0.40 [36%] # (9%)
	21	50	7.57 ± 0.15	9.55 ± 0.79 [33%] # (−9%)	9.57 ± 0.86 [35%] # (−8%)	9.48 ± 0.82 [34%] # (−10%)	9.30 ± 0.79 [30%] # (−2%)	8.34 ± 0.25 [24%] (−0.1%)
	22	100	7.58 ± 0.38	9.03 ± 0.50 [25%] # (−14%)	9.45 ± 0.42 [33%] # (−9%)	9.40 ± 0.43 [33%] # (−11%)	9.42 ± 0.47 [32%] # (−0.4%)	9.03 ± 0.48 [34%] # (−8%)
Acetaminophen	23	50	7.23 ± 0.72	10.54 ± 0.73	10.38 ± 0.62	10.53 ± 0.74	9.45 ± 0.60	8.35 ± 0.36

Notes: Analgesic effect (%) in comparison to (reference drug) and [control]; * Statistically significant (*p* < 0.05) to the group which consumed sodium thiopental; # Statistically significant (*p* < 0.05) to the group which consumed acetaminophen.

**Table 4:** Impact of the *M. chamomilla* dry extract and its amino acid preparations on the duration of thiopental-induced sleep, t ± Δt.

Active Ingredient/Group (n = 6)		Dose (mg/kg)	Average Duration of Sleep (min).	Soporific Effect (%) in Comparison to the Group Given Thiopental	Soporific Effect (%) in Comparison to the Group Given the Chamomile Extract
Control group (1)			0	-	
Gch	2	25	79.67 ± 7.18	75.99	100
	3	50	115.33 ± 12.60	110.02	100
	4	100	76.67 ± 9.91 *	73.13	100
Gch-Arg	5	25	74.99 ± 6.51 *#	71.54	−5.87
	6	50	40.02 ± 5.93 *#	40.09	−65.30
	7	100	50.85 ± 7.55 *#	48.50	−33.68
Gch-Phe	8	25	43.32 ± 8.33 *#	41.32	−45.63
	9	50	54.46 ± 11.12 *#	51.95	−52.78
	10	100	57.77 ± 8.17 *#	55.10	−24.65
Gch-β-Ala	11	25	132.88 ± 7.91 *#	126.76	66.79
	12	50	137.14 ± 3.19 *#	130.82	18.91
	13	100	196.61 ± 11.69 *#	187.55	156.44
Gch-Gly	14	25	73.22 ± 6.10 *#	71.54	−8.10
	15	50	105.76 ± 15.15	100.89	−8.30
	16	100	93.21 ± 12.46	88.91	21.57
Gch-Val	17	25	53.86 ± 5.52 *#	51.37	−32.40
	18	50	61.13 ± 5.99 *#	51.95	−47.00
	19	100	95.54 ± 4.62	91.13	24.61
Gch-Lys	20	25	206.27 ± 8.52 *#	196.76	158.91
	21	50	201.65 ± 6.88 *#	192.35	74.85
	22	100	193.90 ± 14.71 *#	184.96	152.90
Valerian extract (23)		2.15	96.83 ± 8.46	92.37	
Thiopental (24)		40	104.83 ± 8.76	100	

Notes: * Statistically significant (*p* < 0.05) to the group that consumed sodium thiopental; # Statistically significant (*p* < 0.05) to the group that consumed AN NATUREL valerian syrup.

**Table 5:** Physical properties of the aqueous polyethylene oxide (PEO) gels loaded with *M. chamomilla* dry extract, and the corresponding 3D-printed *M. chamomilla* dry extract lattices and discs (mean ± SD, n = 3).

Gels: Extract (g)/10 g	Viscosity, cP (22 ± 2 °C)	Surface Area of the 3D Lattices, mm^2^	S_practical_/S_theoretical_ Ratio	Mass of Lattices, mg	Mass of Round-Shaped Discs, mg
0.5	137,100 ± 9908	362.7 ± 59.9	1.19	129.9 ± 9.1	117.4 ± 1.2
1.0	179,633 ± 9785	456.0 ± 67.4	1.41	149.4 ± 3.4	146.5 ±7.9
1.5	181,767 ± 9887	453.2 ± 68.1	1.40	187.4 ± 7.9	180.4 ± 9.4

## Data Availability

The data supporting the results of this study can be obtained from the corresponding authors upon reasonable request.

## Supplementary Materials

The following supporting information can be downloaded at: https://www.mdpi.com/article/10.3390/ijms25158292/s1.
