# Supplementary material for: German Chamomile (Matricaria chamomilla L.) Flower Extract, Its Amino Acid Preparations and 3D-Printed Dosage Forms: Phytochemical, Pharmacological, Technological, and Molecular Docking Study
Source: Int J Mol Sci. 2024 Jul 29;25(15):8292. doi: 10.3390/ijms25158292 (PMC11311743; doi:10.3390/ijms25158292)
Supplement: Supplementary file 1 [file ijms-25-08292-s001.zip › ijms-3094105-supplementary.pdf]

Table S1. The docking score of phytochemicals' binding at the active site of the selected proteins.

Ligand	5C1M/mu-opioid		1CX2/COX-2		1EQG/COX-1		4KFQ/NMDA-receptor		6B73/kappa-opioid		2CVD/PTGES		6NCF/5-LOX		6X3X/GABAa receptor	
	Affinity (kcal/mol)	CNN pose score	Affinity (kcal/mol)	CNN pose score	Affinity (kcal/mol)	CNN pose score	Affinity (kcal/mol)	CNN pose score	Affinity (kcal/mol)	CNN pose score	Affinity (kcal/mol)	CNN pose score	Affinity (kcal/mol)	CNN pose score	Affinity (kcal/mol)	CNN pose score
4,5-Dicaffeoylquinic acid	-10.19	0.4587	-1.78	0.5513	-1.38	0.4501	-9.45	0.4413	-6.84	0.7377	-8.20	0.3372	-8.06	0.7459	-6.4	0.3781
3,4-Dicaffeoylquinic acid	-10.19	0.4587	-1.78	0.5513	-1.38	0.4501	-9.45	0.4413	-6.84	0.7377	-8.20	0.3372	-8.06	0.7459	-5.6	0.4765
Luteolin 7,3'-diglucoside	-9.29	0.4652	0	0.5548	0	0.4685	-8.05	0.3440	-6.53	0.5480	-9.46	0.3781	-10.85	0.3891	-7.17	0.5276
3,5-Dicaffeoyl-quinic acid	-9.80	0.5466	-2.37	0.5267	-6.4	0.5493	-10.70	0.7496	-5.85	0.6750	-9.54	0.4659	-10.05	0.4505	-7.06	0.5644
Rutin	-11.12	0.3228	0	0.5586	0	0.5043	-5.80	0.4154	-6.83	0.7248	-7.82	0.4735	-8.76	0.4502	-6.56	0.7816
Luteolin-7-O-glucoside	-9.56	0.6739	0	0.4945	-0.38	0.5469	-9.11	0.2525	-2.26	0.6834	-8.45	0.3774	-8.24	0.5702	-5.87	0.6119
Isorhamnetin	-7.99	0.7924	-8.15	0.6803	0	0.6922	-8.53	0.6732	-5.18	0.7515	-9.4	0.6460	-8.52	0.7166	-6.3	0.8264
Luteolin	-8.21	0.8413	0	0.5640	-6.95	0.6118	-4.88	0.4978	-5.57	0.7010	-8.94	0.5414	-8.58	0.7573	-6.47	0.761
Cryptochlorogenic acid	-8.41	0.8107	-3.88	0.5025	-1.41	0.4915	-8.07	0.3818	-5.46	0.6593	-8.07	0.3780	-8.65	0.8774	-6.14	0.3561
Chlorogenic acid	-8.27	0.7236	-4.97	0.4685	-5.99	0.5017	-8.81	0.4930	-5.39	0.7198	-7.72	0.4304	-7.68	0.5863	-5.91	0.6869
Hyperoside	-9.78	0.3398	0	0.4976	0	0.4257	-4.89	0.4491	-5.47	0.7574	-8.1	0.3867	-8.27	0.4721	-6.09	0.7415
Isorhamnetin-3-glucoside	-10.28	0.2467	0	0.5010	0	0.5186	-10.11	0.5157	-5.28	0.6740	-7.56	0.3456	-8.08	0.7620	-6.16	0.7908
Luteolin-4-O-glucoside	-9.50	0.6026	-4.49	0.5279	0	0.6190	-9.59	0.6868	-5.54	0.7177	-8.33	0.3488	-7.89	0.6190	-4.98	0.5845
Neochlorogenic acid	-6.94	0.4411	-5.45	0.4810	-0.66	0.5154	-9.00	0.6977	-5.45	0.6040	-7.01	0.3980	-7.39	0.8060	-5.71	0.5854
Caffeic acid	-5.40	0.8155	-6.89	0.6681	-6.64	0.6885	-7.04	0.7202	-3.76	0.6726	-5.61	0.8203	-5.51	0.7808	-5.61	0.7254
3,4-Dihydroxy phenylacetic acid	-5.53	0.7237	-6.04	0.5821	-6.15	0.7965	-5.84	0.7776	-3.48	0.6418	-5.11	0.7007	-5.41	0.7654	-5.36	0.7053
Vanilic acid	-5.67	0.9025	-5.75	0.6248	-5.64	0.7333	-6.34	0.9128	-3.18	0.6980	-6.46	0.7923	-5.09	0.6891	-4.86	0.7700
Reference ligand	-11.10	0.9215	-11.51	0.9735	-7.78	0.9496	-8.33	0.9830	-6.51	0.8109	-10.28	0.9167	-10.34	0.8341	-7.08	0.8793

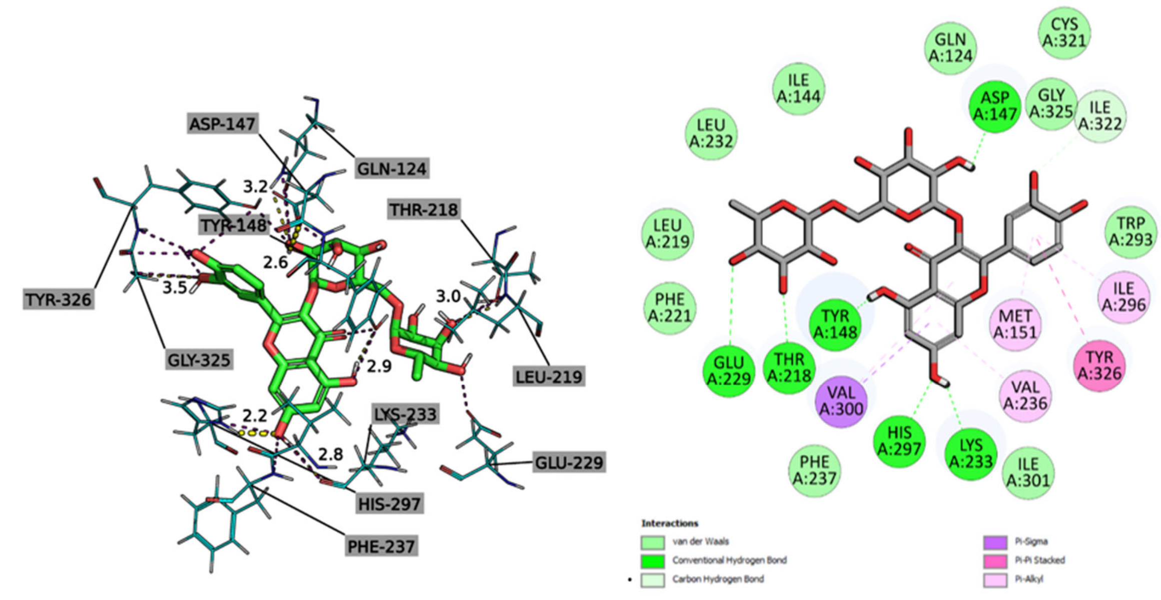

Figure S1.1. Three- and two-dimensional representations of the best key interactions of rutin in the mu-opioid receptor binding pocket. The surface of the protein binding site is surrounded by hydrophobic contacts, mostly π interactions and H-bonds.

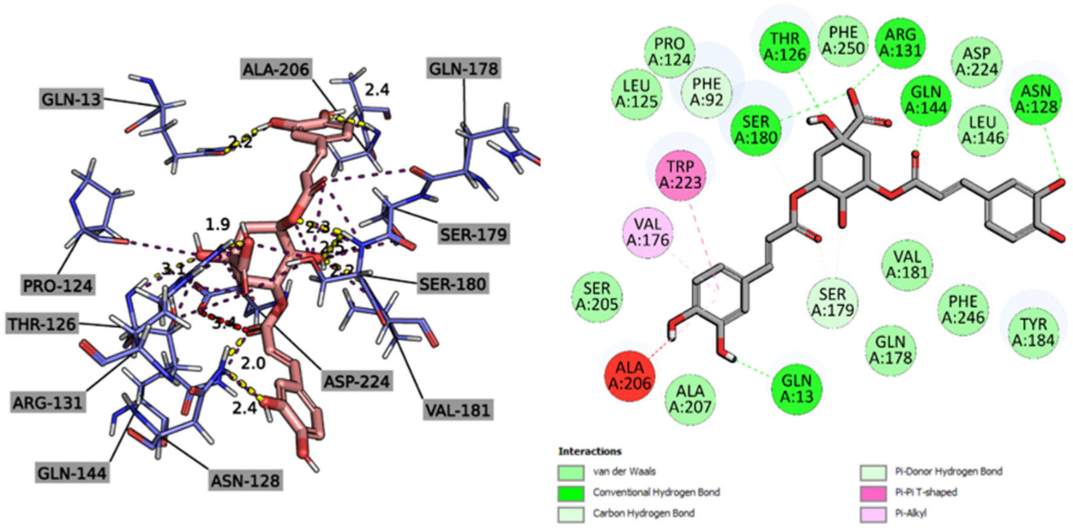

Figure S1.2. Two- and three-dimensional binding modes of 3,5-dicaffeoylquinic acid inside the active site of the NMDA receptor GluN1.

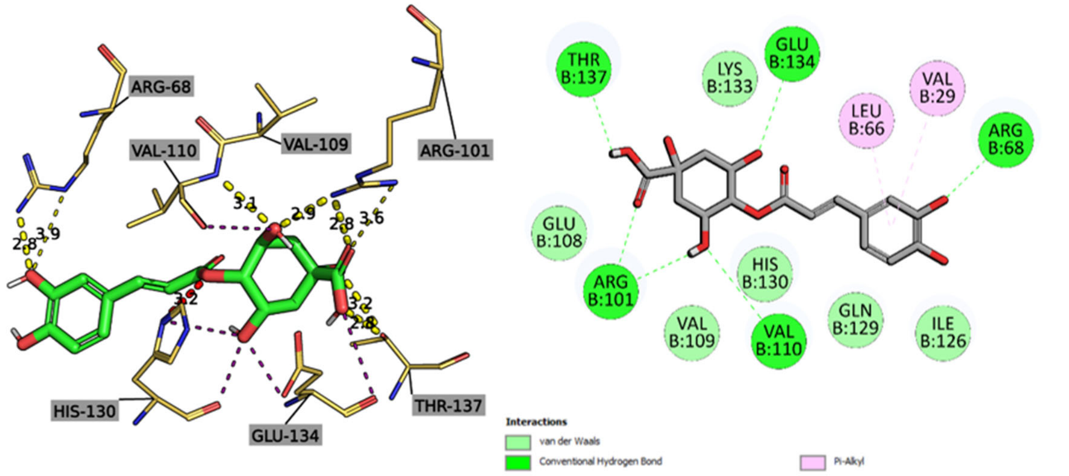

Figure S1.3. Two-dimensional interaction of cryptochlorogenic acid and 5-LOX, 3D image of the surface of the active site of the enzyme.

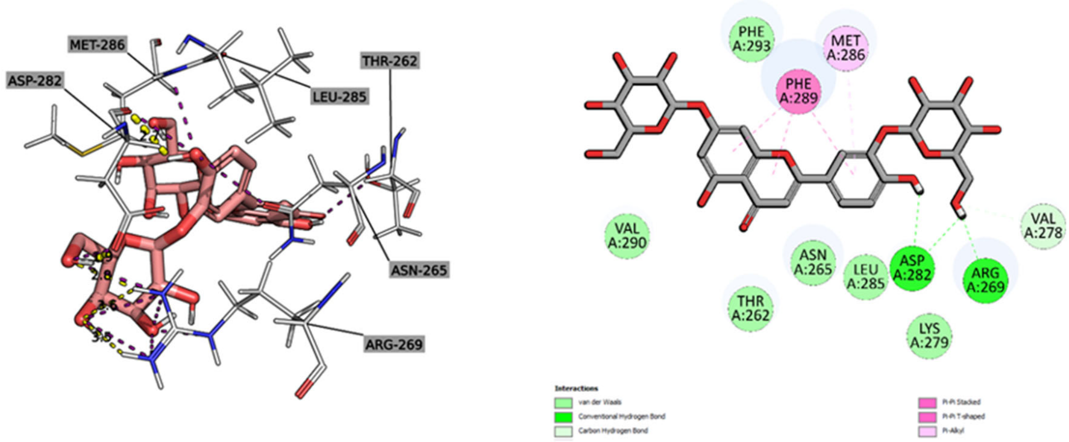

Figure S1.4. Two- and three-dimensional visualizations of the interaction between luteolin 7,3′-diglucoside, and the GABAA receptor.
